# Knowledge and Attitudes Regarding Flatfoot in the Al-Jouf Region, Saudi Arabia

**DOI:** 10.7759/cureus.61842

**Published:** 2024-06-06

**Authors:** Amany Elshenawy, Taif H AlzamIl, Hala M Alkuwaykibi, Ruzan H Alruwaili, Shouq M Alruwaili, Hidayah F Alruwaili, Shuruq A Alruwaili, Anfal S Alruwaili

**Affiliations:** 1 Microbiology, College of Medicine, Jouf University, Sakaka, SAU; 2 College of Medicine, Jouf University, Sakaka, SAU

**Keywords:** saudi arabia, al-jouf region, attitude, knowledge, flatfoot

## Abstract

Background

Flatfoot is a syndrome that includes multiple static and dynamic deformities, characterized by the flattening of the medial arch. It is a common disorder that may affect any age group, causing foot malalignment, pain, and loss of function. Community awareness about flatfoot is essential for the prevention and control of flatfoot complications, ultimately improving the quality of life. The current study aimed to assess community knowledge and attitudes about flatfoot in the Al-Jouf region, Saudi Arabia.

Methods

The study enrolled 315 participants from different sectors of the Saudi population in the Al-Jouf region. An online questionnaire was distributed to them. Answers were scored on a scale of five (never "1", rarely "2", sometimes "3", often "4", always "5"). The level of their knowledge was determined by calculating the scores.

Results

There were significant disparities in the answers, with the majority of participants denying the effect of flatfoot on walking (135, 42.9%), running (123, 39%), standing for long periods (186, 59%), or causing tripping. More than half of the participants agreed that flatfoot never affects school activities or participation in clubs or activities after school. However, most participants showed a good attitude toward individuals with flatfoot, as they were not bothered by their way of walking (199, 63.2%) or how their foot or ankle looks (255, 80.9%), and never embarrassed them because of their foot or ankle (266, 84.5%). Most participants agreed that no one has the right to be unkind to them because of their foot or ankle (276, 87.5%). These positive attitudes are likely due to the cultural norms of the community.

Conclusion

There is a decreased level of awareness about the effects of flatfoot on daily physical activities among the Saudi population in the Al-Jouf region. However, their attitude toward people suffering from flatfoot is highly appreciated. Health education programs are recommended.

## Introduction

Flatfoot, also known as pes planus, is a foot abnormality characterized by a malformed medial arch resulting in abnormal talar plantar flexion and medial rotation, calcaneus eversion, and abduction of the forefoot [1]. This condition is caused by numerous deformities, such as hindfoot valgus and forefoot supination [2]. However, the exact reason for flatfoot deformity remains unclear despite its correlation with multiple factors, including gender, age, weight, and race [3].

The clinical appearances of flatfoot vary greatly between children and adults [4]. In children, flatfoot manifestations range from a flexible, painless deformity to a rigid, painful one. Flexible flatfoot is considered physiological in most children; however, it can lead to early fatigue and medial foot callus. Rigid flatfoot results in discomfort, growing pain, muscle contractures, functional limitation, fatigue, and joint malalignment [2]. In adults, manifestations of flatfoot vary from asymptomatic conditions to ankle pain, bony bumps, and vague pain in the medial foot that increases with activity [4].

Globally, flatfoot is commonly seen and is a significant concern for parents because it can become symptomatic and decrease the quality of life of their children. In Saudi Arabia, there is considerable concern about flatfoot and how to prevent its deterioration through early diagnosis and management. Abdel-Fattah and his coworkers reported a flatfoot prevalence of 5% in 2100 male army recruits in Taif, Saudi Arabia [5].

Prevention and control of flatfoot complications depend mainly on community awareness. Community awareness about flatfoot is an essential constituent of individuals' safety, and it is important to evaluate the different sectors of the community in Al-Jouf to design appropriate health education programs that address their needs. Thus, the current study aimed to assess the community's knowledge and attitudes about flatfoot in the Al-Jouf region, Saudi Arabia.

## Materials and methods

The current study is a questionnaire-based cross-sectional study based on an online distribution of a questionnaire among Saudi citizens in the Al-Jouf region. The study enrolled 315 participants. The inclusion criterion was Saudi citizens in the Al-Jouf region. Individuals less than 18 years old were excluded from the study. The sample was convenient and the questionnaire was distributed through social media applications commonly used in Saudi Arabia.

The sample size was calculated by an online tool (The Survey System Creative, Research Systems), using the following formula (confidence level equal to 95% and the margin of error was 5%) where for 95% confidence level is (1.96)^2^, P is the anticipated proportion of flatfoot in Saudi Arabia, which was unclear and indicated to be 50%, d is the margin of error (5%).

The research proposal was approved by the Local Committee of Bioethics (LCBE) (No.: 1-08-44; approval date 4-4-2023).

The data collection tool involved an open-source and validated questionnaire on flatfoot [6]. The questionnaire includes seven sections: (1) approval for participation, (2) demographic data, (3) questions on physical activities, (4) questions on school and play, (5) questions on emotional effects, (6) questions on footwear and clothing, and (7) questions on other items. Answers to the questionnaire were scored on a scale of five (never "1", rarely "2", sometimes "3", often "4", always "5"). The questionnaire was translated into Arabic and validated by back-to-back translations, and a pilot study was conducted. The questionnaire was distributed through social media applications commonly used in Saudi Arabia.

The data were analyzed using IBM SPSS Statistics for Windows, Version 21 (Released 2012; IBM Corp., Armonk, New York). The results were presented as numbers and percentages. The level of significance was at p<0.05.

## Results

Participants’ sociodemographic data

The current study enrolled 315 participants who agreed to answer the questionnaire. Females showed higher participation (209, 66.3%) than males (106, 33.7%). Regarding age, more than half of the participants were aged between 21 and 30 years old (160, 50.5%), followed by the age group between 31 and 40 years (50, 15.9%), 41 and 50 years (48, 15.2%), 18 and 20 years (40, 12.7%), and above 50 years (17, 5.7%), respectively. About 243 of the participants (77.1%) had a high educational level and were college graduates; however, 72 (22.9%) were students. Marital status showed that 259 (82.1%) of the participants were single and 96 (17.9%) were married (Table 1).

**Table 1:** Participants’ sociodemographic data

Demographic Data		Number	Percentage
Gender	Female	209	66.3%
	Male	106	33.7%
Age (years)	18-20 years	40	12.7%
	21-30 years	160	50.5%
	31-40 years	50	15.9%
	41-50 years	48	15.2%
	More than 50	17	5.7%
Educational level	Secondary	72	22.9%
	University	243	77.1%
Marital status	Single	259	82.1%
	Married	96	17.9%

Participants’ knowledge about the effects of flatfoot on physical activities

**Figure 1:**
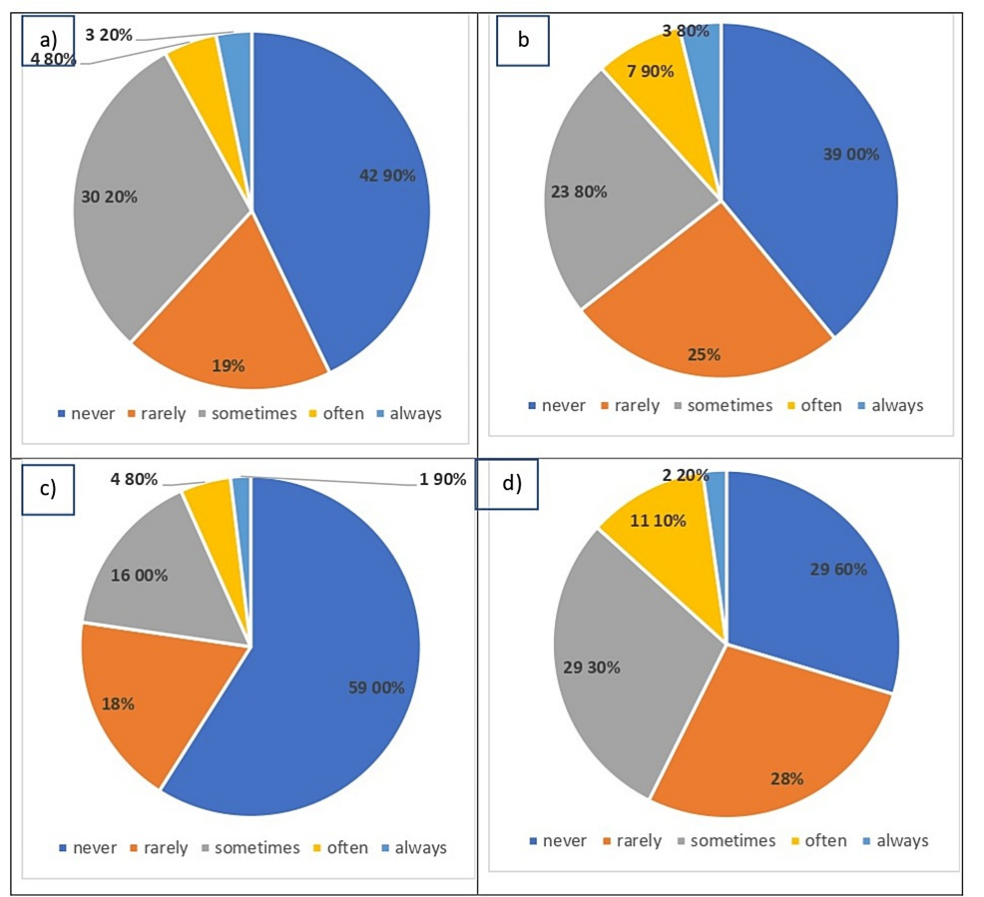
Knowledge about the effects of flatfoot on physical activities, school, and play. Data have been represented as percentages. (a) Answers on the effect of flatfoot on difficulty in walking, (b) answers on the effect of flatfoot on difficulty in running, (c) answers on the effect of flatfoot on difficulty in standing up for long periods, and (d) answers on the role of flatfoot in tripping over feet.

Regarding the participants’ knowledge about the effects of flatfoot on physical activities, school, and play, there were significant disparities. The majority of participants denied the effect of flatfoot on walking (135, 42.9%), running (123, 39%), standing up for long periods (186, 59%), or causing tripping over feet (Figure 1). More than half of the participants agreed that flatfoot never affects school activities or participation in any clubs or activities after school.

Participants' attitudes toward patients with flatfoot

**Figure 2:**
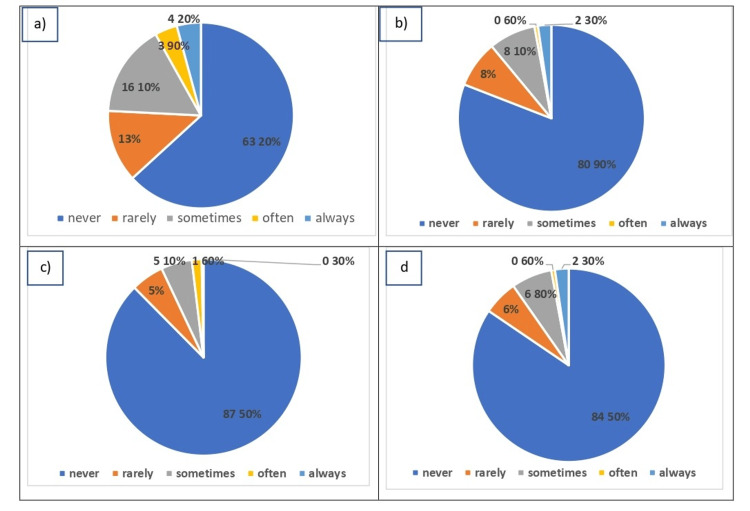
Participants' attitudes toward patients with flatfoot. Data have been represented as percentages. (a) Answers on the attitude toward the way of walking of flatfoot patients, (b) answers on the attitude toward the look of their foot or ankle, (c) answers on the right to be unkind to them because of their foot or ankle, and (d) answers on the emotional effect of flatfoot.

Most of the participants have a good attitude toward patients with flatfoot in their answers, as they never become bothered by their way of walking (199, 63.2%) or how their foot or ankle looks (255, 80.9%), and never embarrass them because of their foot or ankle (266, 84.5%). Most of the participants agreed that no one has the right to be unkind to them because of their foot or ankle (276, 87.5%) (Figure 2).

## Discussion

Flatfoot, also known as pes planus, is a syndrome that includes multiple static and dynamic deformities, characterized by the flattening of the medial arch [1, 7]. It is a common disorder that may affect any age group, resulting in foot malalignment, pain, and loss of function [8].

Flatfoot is a major concern for parents because it could become symptomatic and affect the quality of life of their children. Globally, there is great concern about flatfoot and how to prevent its deterioration through early diagnosis and management [5].

Clinically, flatfoot is described by hindfoot valgus/eversion, forefoot supination with the hindfoot, and a lessened or missing medial longitudinal arch. There are two main types of flatfoot: flexible and rigid. Flexible flatfoot is ubiquitous in newborns and young children and is usually asymptomatic. Rigid flatfoot is generally seen among adolescents and adults, most of which are physiologic or associated with tarsal coalitions, and do not cause pain or functional disability [7, 9]. The main characteristic of flatfoot is the failure of foot-locking during gait [7]. The deformity may be static, improve, or worsen over time [10].

Flatfeet severity ranges from a painless, flexible variant to a stiff or painful tarsal coalition, neurologic disease, or other collagen abnormality [2, 10]. It may be simply a cosmetic concern or result in perceived or real functional deficits due to ankle or subtalar instability [10]. There are no universally accepted criteria for flatfoot [11].

Prevention and control of flatfoot complications depend mainly on community awareness. It is important to evaluate the level of awareness about flatfoot in different sectors of the community in Al-Jouf, to improve individuals' safety and quality of life by designing appropriate health education programs that address their needs.

The current study aimed to assess community knowledge and attitudes about flatfoot in the Al-Jouf region, Saudi Arabia. Results revealed significant disparities, as the majority of participants denied the effect of flatfoot on walking (42.9%), running (39%), standing up for long periods (59%), or causing tripping over feet. More than half of the participants agreed that flatfoot never affects school activities or participation in any clubs or activities after school. However, most of the participants showed a good attitude toward patients with flatfoot, as they never became bothered by their way of walking (63.2%) or how their foot or ankle looks (80.9%), and never embarrassed them because of their foot or ankle (84.5%). Most of the participants agreed that no one has the right to be unkind to them because of their foot or ankle (87.5%). These positive attitudes are most likely due to the cultural norms of the community.

Abdel-Fattah et al. determined the prevalence and risk factors for flatfoot among 18-21-year-old male Saudi military recruits. The estimated prevalence was 5.0%, with common risk factors including family history, wearing shoes during childhood, obesity, and urban residence [5].

In another study in Saudi Arabia, Alsuhaymi et al. [12] assessed the prevalence and risk factors of flatfoot among school-age children. They noted that the highest rate of flatfoot occurred among 7-8-year-olds. In Jeddah, Bourgleh et al. [13] reported that 309 (41.9%) out of 2321 children (88% Saudis, aged under 12 years) had flatfeet.

Almansouf et al. [4] reviewed 361 published studies before July 2021 that estimated the prevalence of flatfoot among the Saudi population and the associated risk factors. They noticed variable prevalence rates associated with age, body mass index (BMI), gender, family history, residence, and the type of footwear worn during childhood.

Regarding the effects of flatfoot on daily activities, in partial agreement with our study, Yasin et al. [14] conducted a questionnaire-based cross-sectional study involving five countries from the Middle East and North Africa region (Jordan, Palestine, Syria, Egypt, and Iraq) to determine the common symptoms of flatfoot, its effect on daily activities, and the use and effectiveness of orthoses. They found that 78.7% of the participants reported that the abnormal appearance of the foot was the most common complaint, 35% complained of severe symptoms that affected their daily activities, and 47.1% had symptoms severe enough to impact their sports or social activities.

The limitations of the current study were mainly the small sample size, online distribution of the questionnaire, and focusing only on one governorate (Al-Jouf region). Thus, further studies on a larger scale are recommended.

## Conclusions

There is a decreased level of awareness among the Saudi population in the Al-Jouf region about the effects of flatfoot on daily physical activities such as walking, running, its role in difficulty standing up for long periods, and tripping over feet. However, their attitude toward people suffering from flatfoot is highly appreciated, as most of them sympathize with these patients and do not become bothered by their way of walking or the look of their foot or ankle. Health education programs on the importance of early detection of flatfoot are recommended.
